# Participant and workplace champion experiences of an intervention designed to reduce sitting time in desk-based workers: SMART work & life

**DOI:** 10.1186/s12966-023-01539-6

**Published:** 2023-11-30

**Authors:** Charlotte L Edwardson, Benjamin D Maylor, Stuart J H Biddle, Alexandra M Clarke-Cornwell, Stacy A Clemes, Melanie J Davies, David W Dunstan, Malcolm H Granat, Laura J Gray, Michelle Hadjiconstantinou, Genevieve N Healy, Panna Wilson, Fehmidah Munir, Thomas Yates, Helen Eborall

**Affiliations:** 1 Diabetes Research Centre, University of Leicester, Leicester General Hospital, Leicester, LE5 4PW UK; 2 NIHR Leicester Biomedical Research Centre, Leicester, LE5 4PW UK; 3 Centre for Health Research, University of Southern Queensland, Springfield Central, QLD 4350 Australia; 4 Faculty of Sport & Health Sciences, University of Jyväskylä, Jyväskylä, FI-40014 Finland; 5 School of Health & Society, University of Salford, Salford, Greater Manchester M6 6PU UK; 6 School of Sport, Exercise and Health Sciences, Loughborough University, Leicestershire, LE11 3TU UK; 7 Leicester Diabetes Centre, University Hospitals of Leicester, Leicester, LE5 4PW UK; 8 Baker Heart and Diabetes Institute, Melbourne, VIC 3004 Australia; 9 Mary MacKillop Institute for Health Research, The Australian Catholic University, Melbourne, VIC 3000 Australia; 10 Department of Population Health Sciences, University of Leicester, Leicester, LE1 7RH UK; 11 School of Human Movement and Nutrition Sciences, The University of Queensland, Brisbane, QLD 4072 Australia; 12 Deanery of Molecular, Genetic and Population Health Sciences, The University of Edinburgh, Scotland, EH8 9AG UK

**Keywords:** Workplace, Sedentary behaviour, Occupational, Benefits, Barriers

## Abstract

**Background:**

A cluster randomised controlled trial demonstrated the effectiveness of the SMART Work & Life (SWAL) behaviour change intervention, with and without a height-adjustable desk, for reducing sitting time in desk-based workers. Staff within organisations volunteered to be trained to facilitate delivery of the SWAL intervention and act as workplace champions. This paper presents the experiences of these champions on the training and intervention delivery, and from participants on their intervention participation.

**Methods:**

Quantitative and qualitative feedback from workplace champions on their training session was collected. Participants provided quantitative feedback via questionnaires at 3 and 12 month follow-up on the intervention strategies (education, group catch ups, sitting less challenges, self-monitoring and prompts, and the height-adjustable desk [SWAL plus desk group only]). Interviews and focus groups were also conducted at 12 month follow-up with workplace champions and participants respectively to gather more detailed feedback. Transcripts were uploaded to NVivo and the constant comparative approach informed the analysis of the interviews and focus groups.

**Results:**

Workplace champions rated the training highly with mean scores ranging from 5.3/6 to 5.7/6 for the eight parts. Most participants felt the education increased their awareness of the health consequences of high levels of sitting (SWAL: 90.7%; SWAL plus desk: 88.2%) and motivated them to change their sitting time (SWAL: 77.5%; SWAL plus desk: 85.77%). A high percentage of participants (70%) reported finding the group catch up session helpful and worthwhile. However, focus groups highlighted mixed responses to the group catch-up sessions, sitting less challenges and self-monitoring intervention components. Participants in the SWAL plus desk group felt that having a height-adjustable desk was key in changing their behaviour, with intrinsic as well as time based factors reported as key influences on the height-adjustable desk usage. In both intervention groups, participants reported a range of benefits from the intervention including more energy, less fatigue, an increase in focus, alertness, productivity and concentration as well as less musculoskeletal problems (SWAL plus desk group only). Work-related, interpersonal, personal attributes, physical office environment and physical barriers were identified as barriers when trying to sit less and move more.

**Conclusions:**

Workplace champion and participant feedback on the intervention was largely positive but it is clear that different behaviour change strategies worked for different people indicating that a ‘one size fits all’ approach may not be appropriate for this type of intervention. The SWAL intervention could be tested in a broader range of organisations following a few minor adaptations based on the champion and participant feedback.

**Trial registration:**

ISCRCTN registry (ISRCTN11618007).

**Supplementary Information:**

The online version contains supplementary material available at 10.1186/s12966-023-01539-6.

## Background

Recommendations around reducing and/or breaking up sedentary time now feature in many national and international guidelines [1–5]. This is in response to the rapidly growing evidence showing that high levels of sedentary time are detrimentally associated with morbidity and mortality [5] and that reducing and regularly breaking up sedentary time has numerous benefits [6], at least acutely. In recent years interventions targeting sedentary time have emerged [7–9], with the desk-based workplace a key context of interest. These interventions have shown potential to reduce sitting time in the short term (up to 12 months). However, with a few notable exceptions [10–12], evaluation of these interventions has mostly consisted of studies with small sample sizes, short term follow-up, and pre-post designs [7].

The SMART Work and Life (SWAL) intervention was designed to reduce daily sitting time in ambulatory desk-based workers [13]. In brief, the multi-component intervention involved organisational-, environmental-, group- and individual-level strategies, with volunteer employees from the target organisations trained to deliver the intervention in order to facilitate scalability. Using workplace champions within an organisation has been shown to be an effective approach to promoting behaviour change [14], with this model also utilised in the wide-scale implementation of the BeUpstanding sit less, move more workplace program in Australia [15]. The effectiveness [16] and cost-effectiveness [17] of the SWAL intervention was evaluated through a three-arm cluster randomised controlled trial (control; SWAL only; SWAL plus height-adjustable desk) with follow-up measures at 3 and 12 months. Findings demonstrated that the SWAL intervention (with and without a height-adjustable desk was effective at reducing daily sitting time [16] and cost-effective from a public health perspective [17]. However, reductions in sitting time were three times greater in the SWAL plus desk group compared to the SWAL group alone. Another key finding was that behaviour change was only observed during work hours, despite the intervention targeting a reduction in sitting throughout the whole day.

Process evaluation in randomised controlled trials has been identified as an important component when evaluating complex interventions [18]. A process evaluation of the SWAL intervention was conducted to help in the interpretation of the quantitative findings [16]. The process evaluation included both quantitative and qualitative methods to gather feedback from participants on their experiences of each intervention strategy and workplace champion experiences of the training and in delivering the intervention strategies. It is anticipated that these data will also provide insight into the facilitators and barriers to behaviour change. Such information will inform any adaptations required to the SWAL intervention prior to wider scale roll out and to the implementation of other similar interventions [19]. The main aim of this paper is to describe the participant and workplace champion experiences of the SWAL intervention. Secondary aims are to (1) explore whether experiences are different across the two intervention groups, and (2) gather insight into the reasons for the lack of behaviour change outside of work hours.

## Methods

### Ethical approval

for the trial was obtained from the University of Leicester’s College of Life Sciences (ref:14,372) and the University of Salford’s Research Enterprise and Engagement ethical approval panels (ref:HSR1718-039). All participants provided written informed consent prior to any study measurements. Recruitment ran from February 2018 to January 2019, with baseline data collected between May 2018 and February 2019 and 12 month data collection from June 2019 to February 2020. The original protocol [13] also included a 24 month follow up but the COVID-19 pandemic and subsequent lockdown in March 2020 in the UK prevented this longer term follow up from taking place.

### Participant and workplace champion recruitment for the main trial

Participants were recruited from six local government councils in three areas of England, UK (Leicestershire (one city and one county council), Greater Manchester (one city and two borough councils) and Liverpool (one city council)). Councils provide services to the local community (e.g., social care, housing, planning, leisure). All councils advertised the study via the staff intranet, in newsletters and with posters. Three of the councils also chose to run presentations to provide more detail about the study. Any staff members who were interested in taking part in the study were asked to complete an eligibility and interest form. Participants were eligible if they were ≥ 18 years of age, worked ≥ 60% full time equivalent, spent most of their day sitting and were ambulatory. The eligibility and interest form also asked potential participants if they would be willing to be trained and take on the role of workplace champion for the intervention (whilst also being participants themselves). In total, 124 individuals selected that they would be willing to act as a champion. There was no specific eligibility criteria for the workplace champion role. Information collected on the interest form was used to group participants into clusters based on their desk location. Clusters were defined by either a shared office space, which could include staff in different teams and departments, or by a team/department located across different office spaces. Clusters were required to have at least one participant willing to take on the workplace champion role. If clusters had more than one participant volunteer to act as workplace champion they could either share the role or decide amongst themselves who would take on the role. Clusters (n = 78 clusters, 756 participants) were randomised to either control (n = 26 clusters, 267 participants), SWAL only (n = 27, 249 participants) or SWAL plus desk (n = 25, 240 participants).

### Intervention

The SWAL intervention is grounded in several behaviour change theories (Social Cognitive Theory [20], Organisational Development Theory [21], Habit Theory [22], Self-Regulation Theory [23] and Relapse prevention Theory [24]) and implemented through the Behaviour Change Wheel (BCW) and the associated COM-B approach [25] The SWAL intervention has been described in detail elsewhere [16], the logic model of the intervention is presented in Supplementary Fig. 1 and the intervention strategies and timing of these are summarised briefly in Supplementary Table 1. The intervention was exactly the same for both intervention groups (SWAL and SWAL plus desk), with the exception of the SWAL plus desk group also receiving a height-adjustable desk and associated guidance booklet (this included the correct ergonomic position when standing and sitting and the recommendations for frequency of posture change whilst using the desk). The intervention content was all pre-prepared for the workplace champions. The workplace champions were responsible for: attending a three hour face-to-face training session to prepare them for the role; launching the intervention (via email and any other methods they chose to use) within their cluster; sending managers the online education link and associated resources; sending a link to the online education to individual staff members; sending out monthly emails (they could tweak the content and format); putting up motivational posters; organising sitting less challenges (some example challenges were given but workplace champions and staff could also design their own); organising and delivering the group catch up sessions (template agendas and content were given); and, acting as positive role models and offering encouragement and support to their group/cluster. Workplace champions were not given any financial incentive for their role (but received £20 vouchers for returning their documentation outlining which parts of the intervention they had facilitated and when) but Senior Management Teams agreed to allow two hours of protected time each month for the champion-related tasks. The individual staff members were responsible for: completing the online education (which included goal setting, barrier identification, action planning and the importance of self-monitoring and prompts); reading the monthly emails; using the self-monitoring and prompt tools; taking part in the sitting less challenges; attending group catch up sessions; and, using their height-adjustable desk and reading the guidance booklet (if randomised to the SWAL plus desk group).

### Measures

The measures were designed to gather feedback and experiences of all intervention strategies.

#### Workplace champion training and intervention facilitation

At the end of the three hour training session, workplace champions were asked to complete an evaluation survey which asked the champions to rate each of the eight parts using a 6-point Likert scale (1 = Not useful at all to 6 = Very useful). All workplace champions were also asked to complete a short questionnaire at the end of the study (i.e., 12 month follow up) which included open ended questions on their opinions on what had worked well/not so well with the intervention components and delivery. At the end of the questionnaire, champions could select whether they were happy to also take part in a telephone interview. All champions that agreed were interviewed.

#### Participant experiences of the intervention

Participants randomised to the intervention groups were asked to complete a questionnaire at 3 and 12 month follow-up which asked closed, open ended and Likert scale questions on their experiences with the main intervention components, i.e., the online education session, the self-monitoring tools, group catch up sessions, sitting less challenges, the height-adjustable desk, as well as questions on any benefits and barriers experienced. A random sample of participants randomised to the intervention groups (and who were still taking part i.e., had not withdrawn by 12 month follow-up) were also offered the opportunity to take part (via email) in a focus group at 12 month follow-up to gather more detailed information on the study and intervention experiences. All participants that were happy to take part, in response to the invitation email, were booked for a focus group. Separate focus groups were conducted for each study arm; all were held in person at the participants’ workplace.

*Topic guides*: *T*o develop the flexible topic guides we drew upon our approach to evaluating previous similar interventions, to explore experiences of delivering and receiving the intervention and factors that helped and hindered engagement. The workplace champion interview guide covered: staff responses to the intervention; experiences of delivering the intervention (including what worked, what did not work, adaptations made); challenges and barriers to delivery; changes made to the workplace; and suggested improvements to the intervention. The focus group guide facilitated discussion on: the experience of the intervention components by the participants (i.e., online education session, group catch up sessions); impact of the intervention on behaviour inside and outside of work; and perceived and experienced benefits of the intervention and barriers to behaviour change.

Interviews and focus groups were conducted by two research assistants (one male, one female) who were part of the study team and had had contact with participants previously through evaluation measures, but were not involved in delivering workplace champion training or the intervention. Both were trained in conducting interviews and focus groups. Their first interviews and focus groups served as a pilot for the topic guide. Interviews and focus groups were audio recorded and transcribed verbatim.

### Analysis

For the forced choice and Likert scaled questionnaire items, frequency counts or means and standard deviations were calculated, all using IBM SPSS v25. Analysis of the qualitative data was conducted by HE, drawing upon the constant comparative approach [26]: a sample of interview and focus group transcripts were read line-by-line to identify initial themes and relationships between them; these were discussed with CE and BM and were converted into an initial coding framework. All transcripts were uploaded to NVivo Qualitative Indexing software (QSR International Pty Ltd. (2012)) and systematically coded. Throughout the coding process, the framework was refined and expanded, new codes added, refining code names and amending code relationships. The responses to the open-ended questions from the participant questionnaires were also uploaded to NVivo to enable coding with the same framework. At a later stage, discussions between HE, CE and BM generated a set of questions associated with the aims of the process evaluation, which informed the addition of further codes which were more deductive in nature. Data coded to each relevant code were retrieved and re-read to identify patterns and ‘weight’ of findings.

## Results

Table 1 summarises the process evaluation data collected to gather participants’ and workplace champions’ views and experiences of the intervention. Supplementary Table 2 reports on the cluster and participant representation in the focus groups by intervention arm and council and workplace champion and participant descriptive data are reported in Supplementary Tables 3, 4 and 5. We subsequently draw from data in Table 1 to illuminate the trial findings: first, how and why they felt the different intervention components helped reduce sitting time at work; then, how their accounts add insight to the lower impact on sitting behaviour outside of work; and finally, cross-cutting themes of benefits of, and barriers to the intervention.

**Table 1:** Summary of the measures

Type of data	Collected from	Timing	Number of participants
Questionnaire to assess workplace champion feedback on training	Workplace champions	End of training	56 workplace champions from 51 clusters (98.1% of clusters represented at training; 1 cluster did not have a champion attend training) were allocated the role of champion and attended the training.
Questionnaire on intervention components	All intervention participants	3 and 12 months	3 months: 180 (78%) SWAL only; 191 (85%) SWAL plus desk. 12 months: 162 (73%) SWAL only; 178 (80%) SWAL plus desk.
Focus groups	> 20% random sample from each study arm	12 months	36 focus groups (14 SWAL only (49 participants, average 3.9 participants per focus group, average duration 45.8 min), 11 SWAL plus desk (51 participants, average 5.8 participants per focus group, average duration 56.8 min)).
Workplace champion questionnaire	All workplace champions	12 months	27 workplace champions (14 SWAL only, 13 SWAL plus desk).
Interviews	All workplace champions who agreed to be interviewed	12 months	16 workplace champions (9 SWAL only (average duration 24.4 min per interview), 7 SWAL plus desk (average duration 29.6 min per interview)) representing 14 clusters (8 SWAL only, 6 SWAL plus desk).

### Workplace champion training

On the questionnaire, workplace champions scored the training session highly, with mean scores for each of the eight parts ranging from 5.3 to 5.7 out of 6. Responses to the free-text items reflected this; champions commented that they “*really enjoyed*” the training session, appreciated the “*helpful information and guidance*” about their responsibilities as a champion and “*good resource materials to take away*,” and valued the opportunity to meet with other champions and share ideas. In the interviews, champions explained aspects that helped them – including helping them prepare and develop more confidence, for example: having a session plan and script as back up, considering the variations in group dynamics that champions may come across; and, helping prepare for different scenarios.

*WPC 101: At the time I was concerned about how to deliver the group catch ups, but [the trainers] covered that with literally giving me a script so that worked quite well.*

*WPC 107: I think that was quite structured within the guidance makeup and setup, if you wanted to know what works well, what doesn’t work well. A lot of that was actually quite nicely done, there was also a good format with that. […] It went through quite a lot of different scenarios for prompting people that might be a bit more quiet.*

Several champions reported that they would have liked the opportunity to role-play a group catch up session within the training session to build their confidence, whilst having the trainer present to provide guidance and feedback. Although this confidence typically developed with practice:

*WPC 105: If you play my first one back compared to my second one you’ll see the confidence in my voice. In that meeting, everybody came out and they were all thrilled to bits. And I felt wonderful because they were all coming and telling me how good it was.*

### Online education

The online education session was rated highly by participants in both intervention groups (see Table 2).

**Table 2:** Overall assessment of the online education session

Statement	SWAL only (N = 161)		SWAL plus desk (N = 161)	
	Agree	Strongly agree	Agree	Strongly agree
The level of the session was appropriate (%)	67.7	19.3	64.6	21.1
The length of the session was appropriate (%)	62.7	12.4	62.1	18.0
The session increased my awareness of the health consequences of too much sitting (%)	70.2	20.5	55.9	32.3
The health consequences covered in the session motivated me to make a change to the time that I spend sitting (%)	61.5	19.3	54.7	24.8
The health benefits of reducing and breaking up sitting motivated me to make a change to the time that I spend sitting (%)	60.2	18.0	55.3	28.6
Overall, the session motivated me to make a change to the time that I spend sitting (%)	60.0	17.5	59.0	26.7

In the focus groups, participants recalled the education content and messages as interesting and clear as well as motivating, reinforcing the questionnaire responses.

*FG17; P3: I thought the little online training thing that we did right at the start, most of the details of which I’ve forgotten, but the message was quite clear, and I thought that was actually really good, really well done, well presented. And a little bit of an eye-opener in terms of the potential health risks. So, that was quite a motivator to get me going on it. (SWAL plus desk)*

Several participants shared the link to the online education with others outside of the study as they felt it was really useful and other participants commented that it should be part of the online learning portfolio for staff.

*FG34; P1: I think it’s fab…. We’ve got an online learning portfolio. And it should be part of the [workplace] induction. Or a tailored version because it’s really interesting. And it’s really professional, informative. (SWAL only)*

Views varied in terms of both the amount and frequency of new online content added during the intervention period, with some participants suggesting providing ongoing content in smaller chunks.

*FG4; P1: I mean, content updated, refresh the content and keep sending the links, and say look, this is something new.*

*FG4; P2: Yes, but do you know something, because we’ve done it once before, and because we’re so busy, I don’t think we would do it again, regardless.*

*FG4; P1: No, but if the content changed, new stories came up, then people would click and see what’s going on.*

*FG4; P3: could you break it down into smaller chunks?*

*FG4; P4: yes, rather than all at once (SWAL plus desk)*

A minority of participants were less convinced that delivering the education via an online platform was the most appropriate method, some commented in the focus groups how face-to-face education provision would have been more preferable and memorable.

*FG26; P1: another e-learning course certainly doesn’t get me excited* (SWAL only).

### Monthly emails

The emails sent by workplace champions had a mixed response. For those who appreciated the emails, some commented that the email content was not so important, rather that they served as a useful prompt to remind or motivate them:

*FG34; P2: What it does do is it reminds you. It does act as a reminder. I’ve never done any of the things and the email could just say, “Stand!” And that would probably do as much good for me as everything that’s in it because that’s the only thing it’s doing. It’s just giving me a prompt to remind me. (SWAL only)*

Others emphasised the need for different content, to enhance interest:

*FG29; P1: I think the email is good. But more from your point of view, making sure you’ve got a suite of a different kind of tasks or things, so it doesn’t become repetitive. (SWAL only)*

Champions reported doing their best to stick to the email schedule, although sometimes fell behind when their workload was high. Some felt that the emails provided by the champions were too short or simple and would have benefited from livening up with animations or including links to useful sources, such as publications of relevant studies. Indeed, some champions adapted and personalised the emails, which their group members appreciated.

*FG33; P1 (WPC): Yes, last time I had to make up quotes for them. Because we didn’t have time [to get quotes from members to include].*

*FG33; P2: Apparently, I said I sold my couch because I don’t need it anymore! […]*

*FG33; P1 (WPC): I always put some nice pictures in. Things like that. People stretching and clocks. Because it’s ‘do your time’. (SWAL plus desk)*

Other participants talked of ever-increasing email traffic and overflowing inboxes with their everyday work, meaning “email blindness” and that SWAL emails just “get lost” (FG28 SWAL only).

*FG20; P3: The email prompts, I mean, a lot of people, most people get a lot of emails I’m guessing every day. So, they probably read it and go, oh okay and then onto the next thing. (SWAL plus desk)*

As well as ideas for adapting the emails, alternative suggestions included: screensaver messages and pop-up messages a few times a week (such as IT messages) on Yammer or Microsoft Teams (note: this was pre-Covid-19 when Microsoft Teams use was less common).

### Group catch up sessions

A high percentage (> 70%) of questionnaire participants agreed/strongly agreed that the group session helped them formulate plans to sit less, motivated them to sit less and that meeting as a group (rather than individually with the workplace champion) was worthwhile (Table 3). A slightly lower percentage agreed/strongly agreed that the sessions helped them to find solutions to any barriers and stay on track with their plans.

**Table 3:** Participants’ experiences of the group catch-up sessions

	SWAL only (N = 157)		SWAL plus desk (N = 175)	
Statement	Agree (%)	Strongly agree (%)	Agree (%)	Strongly agree (%)
These sessions helped me formulate plans to sit less	64.5	6.4	58.4	9.7
These sessions helped me stay on track with my plans to sit less	55.5	6.4	58.8	6.1
These sessions motivated me to sit less	66.4	8.2	61.4	9.6
These sessions helped me find solutions to barriers I have experienced	46.7	6.5	43.8	5.4
Meeting as a group (rather than individually with the Champion) was worthwhile	66.4	10.3	58.9	17.0

The focus groups helped to explain the questionnaires responses; many participants spoke positively about the catch-up sessions, describing how the group setting was useful for sharing tips and staying motivated.

*FG4; P1: Definitely useful. Like emails, as I said, if you keep refreshing, it’s motivating. Sometimes people lose track […] so you help people to get back on track […]*.

*FG4; P4: It’s just good to hear other people’s techniques about what they do and get their ideas about how they move and carry out their day and things, so that was useful. (SWAL plus desk).*

Participants highlighted that the catch-up sessions were an opportunity to reflect (which they did not usually have a chance to do), share ideas and discuss barriers experienced.

*FG34; P2: It was good to think about and talk about it. And have a space to just come away from your work and consider how, actually, you can start making it a part of your routine, a bit more. Because [we’re] just into our habits and we don’t really have a chance to reflect. […]*

*FG34; P1: It was amazing. It was like a TedTalk […] If nothing else, it’s good for those who are brave enough to come along and fess up about how they’ve been doing or haven’t been doing. I think it’s a good reminder of why you’re part of it. Getting that information […] and some coaching to help you think through what the barriers might have been. (SWAL only)*

Many champions agreed, and noted the benefit of participants collectively deciding on challenges in these sessions.

*WPC 111: Just getting people to chat about what they’ve done, what they’ve found useful, what they haven’t managed to do, share ideas for what each other could do. And then almost that moral support. So you can say, ‘yes I was too embarrassed to do this’ or ‘it was like this when I tried that, why don’t you try that?’ And then sharing ideas to do a challenge, because each time we’ve done a [catch-up], we’ve come up with a different challenge afterwards that we’ve all shared. So I think everyone’s come out of that feeling much more motivated.*

Furthermore, some participants talked about how the sessions created a sense of being a team and group support and encouragement, which had an impact over and above supporting behaviour change.

*FG6; P4: And also encourage others so it’s like, you’re not alone, we’re in it together.*

*FG6; P2: It has given us an element of solidarity really which is quite nice. […] it’s almost like the ten or 12 of us have got something in common that’s just for us. (SWAL plus desk)*

Those that found the sessions less helpful explained this as due to the repetition of content, but also noted how the group make-up dynamics could impede a session’s success.

*FG26; P1: I think the first one was okay. I think the second one there was just a lot of repetitive group discussion. We just covered the same stuff and I think the characteristics of the team and the teams on this level could be quite different from other teams. So, a lot of the people we work with are maybe quite introverted so not as outspoken so you don’t get the same openness to discuss. (SWAL plus desk)*

And a few participants and champions commented that the catch-up sessions felt less necessary for them, given their close proximity in the office to other participants, meaning they regularly discussed sitting less.

*WPC 113: We’re only a small group anyway, so when we get the catch ups, sometimes you just think, is it a waste of time in some respect? Because we know we motivate each other but we do [the session] […] We live and breathe in that same office. So we sit and have a chat about what we’re doing, you know? How we’re feeling and so on.*

Adapting the session to their group’s make-up and circumstances was something several champions talked about, for example if they felt that the ‘script’ would not be well received by their group.

*FG27; WPC: The thing about the group catch-ups is, you get like a six-page script as a champion that you’re supposed to do. […]Our team, we’ve all been there years, and we’re very close. If I sat there with that script, they’d absolutely laugh me out of the room. (SWAL only)*

Workplace champions commented on the logistical difficulties of “*rallying people together*” (WPC 110) for the sessions due to both their own and their group members’ workloads (WPC questionnaire), differing work schedules, patterns, and commitments, and the acknowledgement that “*only certain people who turn up*” (WPC 116). Despite the challenges, champions typically felt that the sessions helped remind and motivate participants.

*WPC 116: [if] you don’t have these catch-up sessions, people just would forget what they’re doing, or think ‘don’t bother’.*

Indeed, some participants commented that it would be helpful to meet as a group more frequently, but recognised the difficulty in getting the balance right.

*FG29; P1: But I wonder […] whether it would be better to meet more frequently, just to remind everybody that it’s what you’re doing and that it’s … still important. […] It’s a hard balance, isn’t it, between being too much and not enough. (SWAL only)*

### Sitting less challenges

The response to the sitting less challenges was also mixed; approximately 60% and 50% of SWAL only and SWAL plus desk participants respectively agreed or strongly agreed that they enjoyed the challenges and the challenges motivated them to sit less and reduce their sitting time (Table 4). This mixed response was reflected in the focus groups; some participants commented that the challenges were fun and a good way to “*get people energised*”, noting the enjoyable competitive element within and between teams.

*WPC 105: When we’ve done the competitive stuff […] there’s been quite a bit of fun, banter and you know, that’s quite nice. […] A lot of us have worked together for a long time, and […] you can have a more light-hearted approach to it,[…] So when we did the competitive element, and you know, ‘oh gosh, so and so… you’re last in terms of the competition’. You have to know people well to take it to that level really so that’s been good.”*

Others preferred to ‘’*get on with it’’* themselves or felt challenges were difficult to engage with if they did not have a device to record their steps.

*FG30;P1: I think I was just more interested in, more naturally interested in just getting on with it myself, making myself do it […] I did intend to actually record it and join in the challenges, but it petered out. (SWAL only)*

*FG7; P2: Unless you’ve got a device to monitor your steps, people aren’t going to do it because they haven’t got the devices to do it, and it’s very time consuming. (SWAL plus desk)*

**Table 4:** Participants’ feedback on sitting less challenges

	SWAL only (N = 152)		SWAL plus desk (N = 172)	
Statement	Agree (%)	Strongly agree (%)	Agree (%)	Strongly agree (%)
The challenges increased my motivation me to sit less	50.0	14.5	41.9	11.3
The challenges reduced my sitting time	46.8	12.9	45.9	8.2
I enjoyed the challenges	53.2	11.3	41.9	6.5

The slightly lower percentage seen for the SWAL plus desk group was reflected in some of the focus group comments, where participants felt that the challenges may have been more useful for reducing sitting time for the participants who did not receive the height-adjustable desk.

*FG20; P4: I think it would be interesting to see the difference that the workplace champion intervention has had on a group that don’t have the desks. […] Because we’ve got the desks to motivate us on a daily basis as a physical, it’s there.*

*FG20; P2: If I didn’t have the desk, I’d just be like…*.

*FG20; P4: If all you’ve got is that email and that activity challenge …*.

*FG20; P1: The value of the email and the challenges will be heavier for those without desks.*

*FG20; P3: I think it would probably take longer to instil those changes you know, because the desk is the most simple and, I think, positive solution to getting people more active. (SWAL Desk)*

A particular difficulty, in both intervention arms, was maintaining engagement in the challenges over time as the novelty wore off or work took priority.

*FG27; P2: I think perhaps though, the challenges, probably we’re like, “yes we’ll do a challenge.” And then you get back into your work routines, and then work takes over. (SWAL only)*

To address this, champions gave examples of efforts to re-motivate participants, for example adding in a charity donation, incentive or adapting the challenges to make them more suitable or relevant, through discussions with individuals in their group.

*WPC 109: It’s a talking point as well half the time […] we’ve done the Land’s End to John O’Groats challenge which actually swiftly got changed to Land’s End to Liverpool, because […] we’d still be doing that till way past the end of the project. So exactly, that’s been quite good […] we did an update from Google Maps and put who is where on the map, in terms of walking to try and generate a bit more competition.” (SWAL only)*.

### Self-monitoring and prompts

Participants who used the self-monitoring and prompt tools commented how the tools increased their awareness of sitting for long periods, encouraged them to take breaks and highlighted that they were not as active as they had thought.

*“I notice I really didn’t take any breaks, so it does prove as a good prompt. Even if you only take a break (for) a percentage of the prompts it’s still useful.” (Questionnaire; SWAL plus desk).*

*FG11; P1: It makes you aware. You know, you’re sat- you’re doing a piece of work and you think you’re only there for five, ten minutes. And then the thing goes off to say actually you’ve been sat for 30 min. So, it does give you that sort of prompt to get up and move around. (SWAL only)*

*FG26; P1: And I think of myself as pretty active but the walking app that I’ve put on my phone […] told me I wasn’t. So, it really showed me how inactive I was in terms of walking when I thought I was more active. And it also showed how the smaller things can get your steps up that you don’t really think about but are pretty easy to incorporate. (SWAL plus desk)*

Others reported finding the tools frustrating and distracting; while some continued using them despite this. Others turned them off as they interrupted their workflow and often popped up at inconvenient times.

*FG4; P2: I’ve got the WorkRave thing, and when I first had it, I did tend to do some of the exercises and things. […] Partly I think it’s because my workloads just got more intense recently, but now when it pops up, I shut it down. It’s just become a thing that, it’s just a distraction really. (SWAL plus desk)*

*“At one point I got frustrated about being interrupted […] when I had a deadline to achieve. [But] I was quickly reminded of the shooting pains I used to get across my shoulders when I had worked all day - I have kept the app on ever since.” (Questionnaire; SWAL only)*.

Many participants talked about using their own methods or wrist-worn devices, such as Fitbits, and often reported that as a result they did not use the other suggested tools and felt that these were easier to use as it did not require them to be carrying or looking at their phone or computer.

*FG1; P1: I’ll tell you what we did do. […] we set up a spreadsheet.*

*FG1; P2 What did it capture?*

*FG1; P3 It’s easy. Your day broken up into half an hour slots and you just put a one or a two as to whether you’ve stood or not. And that for me…*.

*FG1; P1 It works out the average day for that day, we’ve got weekly average, running totals. We do work in business intelligence.*

*FG1; P3 A bit of competition. But that really helped me to try and maintain it, maintain an average, don’t let it drop too much and that kind of thing. Good thing, there’s none of the apps that I… I don’t know, none of them worked or whatever. That has been my main recording and knowing what I’ve been doing. (SWAL plus desk)*

*FG35; P1: I haven’t really used any of them because I’m not someone who uses loads and loads of apps. But I’ve got a Fitbit, and I’ve had a Fitbit for probably a year and a half, two years maybe. So […] I don’t really have room for another thing (SWAL only).*

### Height-adjustable desk

Many SWAL plus desk participants felt that having a height-adjustable desk was key in changing their behaviour and enabling them to take breaks in sitting without having to leave their desk.

*FG7; P1: If we didn’t have the desks, then I don’t think it would have made any difference to me [in changing habits], to be honest with you. I think having the desk made a huge difference to me. (SWAL plus desk)*

Participants varied in their experiences on what tasks were suitable to do standing up; task-related factors were reported as influential for some questionnaire respondents (Table 5). Many participants felt that phone calls and emails were well suited to standing, but tasks such as more substantial written work were difficult to complete standing up.

*FG17; P2: It’s easier with emails. It’s harder for writing a report or doing any real written work. If I have to write a lot of reports, I find it’s difficult to do that. (SWAL plus desk)*

Relatedly, participants varied in terms of how they used the desk for tasks requiring concentration, with some struggling to concentrate when standing up and others finding that standing up to improve their concentration.

*FG6; P4: I can work a bit better sitting down, being able to really get into a piece of work as opposed to standing up. I’ve not been able to find the sweet spot when standing. I can find myself, when I am standing, focusing more on trying to be comfortable while standing than actually on my piece of work. So, I immediately go, well I’m not concentrating on my work, let’s get back to the sitting down again. (SWAL plus desk)*

*FG7; P2: I think when we first started, if I had something I had to concentrate on, I thought I had to be sat down and hunkered over it. Whereas actually I’m better, now concentrating on those tasks while I’m stood up and it’s just a little bit of a mindset change. I don’t have to be hunkered over the desk and, you know, like that. I can do that stood up. I can work through the complicated stuff whilst standing just as well as I can while sitting. (SWAL plus desk)*

Indeed intrinsic factors (such as learning what works for oneself) was a high reported influence on desk use in the questionnaire (Table 5), as were time-based factors.

**Table 5:** Factors influencing desk usage in the standing position

	3 months (N = 182)		12 months (N = 161)	
Factor	Often (%)	Very often (%)	Often (%)	Very often (%)
Task-based factors (e.g., reading emails)	23.2	7.7	21.8	6.4
Time-based factors (e.g., in the afternoon, every hour for a certain length of time)	36.3	21.4	31.3	15.6
Prompt-based factors (e.g., when the phone rings, when someone comes to see you)	11.0	6.6	13.5	2.6
Intrinsic factors (e.g., when your body tells you it’s time to stand up/sit down)	48.3	24.2	37.9	25.5

In terms of time-based factors, participants shared examples of incorporating desk-use into their working day routine, such as using it in the morning when first arriving at work to start the day standing or after lunch to help with digestion or the post lunch slump.

*FG20; P1: I’ve just changed my routine now. I have a 30-to-40-minute drive in […] so I don’t even sit down when I come in. I make a drink, put the stand up and my first least hour in the day is stood up, unless I feel tired. (SWAL plus desk)*

Although not as frequently reported in the questionnaire, the influence of others was a key theme throughout the focus groups.

*FG6; P3: And what’s helped me is because colleagues in the same desk block have got [desks], it’s encouraging me when I see the others do it [stand] as well so that’s helped me. It’s not just the prompts. It’s seeing the others do it.*

*FG6; P1: You do feel guilty, don’t you? I used to sit next to [name], and he’s really good and he’d always be standing and I’d think, ‘oh I’m not standing’.*

*FG6; P3: ‘I’d better do it.’ (SWAL plus desk)*.

*FG26; P1: That’s the way it’s worked on our level. It’s the meerkat effect when someone pops up and you go, oh yes! (SWAL plus desk)*

However, participants also explained barriers to using the height-adjustable desk, including a lack of space on the desk, difficulties with operating the desk and less normative pressure.

*FG25; P1: I don’t use it as much as I first did or as much as I thought I would use it. And partly, as someone mentioned before, it’s about having bits of paper and other things that you need when you’re standing up, it doesn’t work very well with that. And my desk was, until yesterday, an absolute tip. So, it’s a bit awkward because you get some stuff stuck underneath it. (SWAL plus desk)*

*FG16; P5: I’ve had to stop using it as you know because I find… I don’t know if it’s because I’m petite or small, lifting it was straining my back. I have upper back problems anyway and then I found that aggravated it a bit. (SWAL plus desk)*

*FG26; P1: I think overall people have taken to it, but I think in the last six months the meerkat effect has stopped. People are actually finding their desk a little bit clunky, a bit space consuming for other stuff […] actually doing other things on your desk is quite limiting with these yoyo desks. Trying to write something, I have to move and I’m interfering on the person next to me. (SWAL plus desk)*

### Reducing and breaking up sitting time outside of work

Many participants discussed changes they had made to their behaviour outside of work, which included standing or moving around for the activities they usually did sitting down (e.g., taking phone calls, playing on the games console, watching TV, chopping food, waiting for the train, using the laptop), joining a gym or exercise class (e.g., yoga) or walking more.

*FG4; P2: ’I think it’s not just at work. I think even at home I’m consciously now, if I’m talking to somebody in the past, I would sit for half an hour, but now I’m actually walking, I’m pacing up and down, whilst I’m doing that. Or, sometimes, even if I’m watching TV, I’ll just stand and watch a little bit. It’s not just at work, I think it’s made an impact on my whole day’’ (SWAL plus desk)*.

However, participants typically mentioned these were small changes, which aligns with the trial findings that significant behaviour change was only reported *during* work hours. Indeed, in the questionnaire, “too tired” was commonly provided as a barrier for sitting less at home and some focus group participants admitted that they sat more at home *because of* standing more at work.

*FG17; P3: Definitely at work, probably not much effect at home, to be honest. I’m just aware of the fact that I spend way to long sitting because I spend half my time at home, playing on the PC and you can’t stand up very easily doing that. So, I still, unfortunately, fall into that, but definitely at work. (SWAL plus desk)*

*FG3; P1: ’The opposite is I’ve been standing at work today so I’ve done really well, and I have another chunk of chocolate bar or not doing anything at home. I’ll sit and watch telly for a bit longer because I’ve done what I think I should have done. (SWAL plus desk)*

Others felt that they were already “on their feet” or active after work so could not reduce sitting any further.

*FG7; P2: Outside of work? Personally I can’t [sit] a lot less because I’ve got dogs, so I’m up at six in the morning, walk for an hour, get here, stand for four hours, you know, blah, blah, at least four hours. […] Then I get home and again, dogs, feed dogs, cook my dinner, so by the time… I probably don’t get to sit down until eight o’clock at night. […]. So the longest period I can get to sit down is two hours. (SWAL plus desk)*

### Benefits of sitting less

Data from both the questionnaire and focus groups showed that participants from both intervention groups reported benefits from taking part in the intervention. The majority of benefits mentioned centred around feeling less fatigued and having more energy, with some mentioning this was helpful in the afternoons following lunch.

*FG21; P1: I think going out at lunchtime for our walks has definitely… It makes the afternoon easier. I feel a bit more motivated and a bit more energised in the afternoon on the days you go for a walk. (SWAL only)*

*‘’More energetic; better clarity of thought; more alert; better mind ‘’ (Questionnaire; SWAL plus desk)*.

Many participants felt that the reduction and breaks in sitting had a positive impact on their focus, productivity, ‘alertness’ and concentration.

*“It helps me to focus more on a task. I felt less fatigued at the end of the day. I feel more alert and productive.” (Questionnaire; SWAL only)*.

*FG25; P3‘’But the other thing that most prompts me to stand is when I’m deep into something and I’m struggling to concentrate, I actually just find standing up is like a little bit of a refresh. And that’s the thing that makes me mostly want to stand, is actually when I’m, I’ve really got to get something complete and it’s challenging, mentally. I feel like standing and it helps me to do that” (SWAL plus Desk)*.

One key difference between the benefits highlighted by participants across the two intervention groups was that the group that received the height-adjustable workstation commented that reducing sitting had helped to alleviate previous musculoskeletal problems and relieve general aches and pains. Here, participants mentioned benefits to the neck, shoulder, back and hips.

*“Previous aches in neck and hips reduced; more comfortable standing.” (Questionnaire; SWAL plus desk)*.

*FG7; P2: I think it was helpful for the back from sitting. I used to get loads of just achiness and back problems from just sitting all day long, you know, at your desk, so positive from moving around. […] The aches and pains have gone away. (SWAL plus desk)*

### Barriers to sitting less

Barriers to sitting less or moving more that were mentioned in questionnaire responses could be grouped into: work related; interpersonal; personal attributes; physical office environment (including lack of height-adjustable desk for those in the SWAL only group); and, physical (tiredness and aches). These were also highlighted in the focus groups.

Workload and work and time pressure were the most commonly reported barriers to reducing and breaking up sitting, with many reporting not feeling able to reduce and break up sitting with urgent work or deadlines or forgetting to break up sitting when engrossed in a task.

*“Business of work - we have been overwhelmed and understaffed.” (Questionnaire; SWAL only)*.

*“Feeling tired/stressed/ too much on and no band width to think of the standing bit!” (Questionnaire; SWAL plus desk)*.

*FG6; P2: I think I’d got to a point where I was standing a lot. But I noticed that when things changed for me in terms of work… So, at the moment we’ve got a huge project that we’re trying to get over the line, quite stringent deadlines, really overwhelming with a lot of work. I’m not standing at all. […] I’m just sitting all the time. And I do miss the standing. (SWAL plus desk)*

Needing to concentrate on a task was noted earlier as a factor influencing desk use; this was also mentioned by some SWAL only participants.

*“Need to concentrate for long periods in my job makes regular standing difficult.” (Questionnaire; SWAL only)*.

Another barrier commonly reported was the frequency and length of meetings, which participants found uncomfortable standing in – particularly those with either senior staff or external colleagues who were not part of the programme or concerns about others’ perceptions of it being “strange” to stand up. Many also reported that the workplace is designed for sitting.

*‘’Feeling self-conscious - during certain meetings involving senior management. I am less comfortable standing. I am ok with this during ordinary team meeting’’ (Questionnaire, SWAL plus desk)*.

*‘’Standing in meetings is not culturally acceptable (I sit for 3 h in meeting without moving). No high tables at work- nowhere to stand to look at the laptop or documents’’ (Questionnaire, SWAL only)*.

Similarly, some participants mentioned feeling self-conscious about being the only one standing at their desk or about others’ perceptions of them spending too much time away from their desk.

*“I do feel conscious if I keep getting up as it may give an impression that I am not working.” (Questionnaire; SWAL only)*.

*FG11; P3: I’d also look a bit of a lemon because there’s nobody else standing around me. If I answer the phone with the headset on and I’m chatting away, I’m above everybody else. (SWAL only)*

The lack of a height-adjustable desk for participants in the SWAL only arm was mentioned as a key barrier to reducing sitting time whilst working and a few reported that they had tried to stand at their standard desk, but this led to neck, back or foot ache, hence impractical. Many also said that it was difficult to take time away from their desk to reduce and break up sitting or that there was nowhere suitable to place their laptop to stand up and use it.

*“Not having anywhere to position my laptop to be able to work standing up.” (Questionnaire; SWAL only)*.

*‘’Desk not practical i.e. it is too low to work at the computer. Phone - handset doesn’t stretch very far’’ (Questionnaire, SWAL Only)*.

Finally participants admitted to being “lazy” or forgetting.

*FG26; P1: Some kind of ingrained, just laziness. I’ll be sat down, and I just think just get up and I just go ah, carry on. (SWAL plus desk)*.

*“Just forgetting to move sometimes.” (Questionnaire; SWAL plus desk)*.

## Discussion

The randomised controlled trial demonstrated that the SWAL intervention (with and without a height-adjustable desk) led to lower sitting time compared to control, although reductions in sitting time were three times greater in the SWAL plus desk group compared to SWAL alone. The data presented here highlight that the overall experiences of the SWAL intervention (i.e., the elements that were the same across intervention groups) by workplace champions and participants were positive. While it is evident from the process evaluation data presented that there was variability across the participants in how elements of the SWAL intervention were experienced, there appeared to be no major differences between the two intervention groups in their experiences of the non-desk elements, suggesting that the larger reductions in sitting time observed for the SWAL plus desk group could be attributed to the height-adjustable desk. Moreover, the data on champions’ and participants’ experiences help to explain how the different intervention components helped, notably *how* the addition of the height-adjustable desk helped more than the intervention alone, as well as factors that made each element challenging. The data provided insight into why reducing (or further reducing) sitting time at home was more of a challenge, including tiredness after work, perceiving time after work as relaxation time and/or already feeling sufficiently active.

Similar to our previous study, SMArT Work [27], the education session appeared to be key in increasing awareness of the health consequences of sitting too much and motivating the participants to make a change. However, although engagement with this aspect was high and the feedback on the session was largely positive, the percentage of participants agreeing or strongly agreeing to the statements was slightly lower than a previous face-to-face version of the education session [27], suggesting that face-to-face delivery may be more impactful. During the intervention design process we conducted patient and public involvement work to ensure the intervention was fit for purpose and this suggested that workplace champions would not feel comfortable delivering the education content and that logistically organising face-to-face sessions would be challenging and time consuming. Correspondingly, it was anticipated that face-to-face education would likely result in lower engagement with this intervention component, particularly given the larger sample size in the current study compared to SMArT Work [11]. The decision was made therefore to translate the face-to-face session to online delivery, a method of delivery which has very much now become the norm as a result of the COVID-19 pandemic. Several participants felt that the online education was interesting and motivating, with some suggesting that it would useful as part of staff induction or as part of their online mandatory training portfolio. Based on the feedback, future versions of the SWAL education component could include delivering the education in smaller chunks over a longer period of time or regularly adding some new content to act as a refresher and reminder.

The experiences of the group catch up sessions and sitting less challenges were more varied and mixed, with approximately two thirds finding them useful and approximately a third of participants not. Some champions adapted the sessions and challenges to the needs and circumstances of their group and/or found more buy-in from their group when the group were involved in collectively coming up with the challenge. Going forward, in order to build champions’ confidence to be adaptable, the training for the champions may need to include examples of how they could do this. This could be incorporated using role-play given the champions mentioned that this would have been a helpful aspect to the training to build their confidence for delivering the group catch up sessions.

Despite evidence indicating the importance of self-monitoring and prompts for behaviour change [28, 29] participants attributed lack of use of the prompting tools provided to time pressures or a perception that the strategy would not be useful for assisting them in changing their behaviour. When deciding on self-monitoring and prompt tools we wanted to suggest ones that were free to use to improve the scalability of the intervention in the future. We also wanted to provide the participants with options as we have previously found that one tool is not suitable for everyone [28]. Unfortunately, there are a limited number of tools available that specifically focus on self-monitoring of sitting and prompting breaks in sitting. A range of mobile phone applications and computer software were suggested to participants along with detailed user guides. Some participants did report finding these useful for increasing awareness of the need to take breaks in sitting but some participants felt that they were more of a distraction and found them annoying. Many of the suggested computer software and google chrome extensions required specific approval and installation by the organisation’s IT team, which, for some, created logistical issues and a barrier. Many participants already owned or specifically purchased a wrist-worn commercial device (e.g., Fitbit) and reported that these were easier to use rather than looking at a phone or computer. Though these devices are largely focused on self-monitoring physical activity rather than sitting time, participants did comment that the vibration function within these devices was useful for prompting them to get up. Given the increasing uptake and sophistication of wearable devices [30], future research could further explore leveraging participants’ own devices for monitoring and evaluation. For individuals who do not want to use technology (i.e., wearable devices, computer software, mobile phone apps), our results suggest that encouraging individuals to use time based (e.g., every hour) or intrinsic (e.g., when my body feels like it needs a change in posture) factors for promoting a change in posture may be useful.

Although participants’ experiences of using the height-adjustable desks varied i.e., on the tasks they could do whilst standing and factors influencing use in the standing position, there was high consensus amongst the SWAL plus desk participants that having access to the height-adjustable desk was key to them changing their sitting behaviour. Given the results of the process evaluation indicate that experiences of the non-desk intervention strategies were similar across intervention groups (i.e., SWAL and SWAL plus desk) the larger reductions in sitting time observed in the SWAL plus desk group in comparison to the SWAL only group (-63.7 min/day vs. -22.2 min/day respectively) [16] could be attributed to the desk.

Our trial results showed no significant changes in sitting behaviour outside of work [16]. Many participants in the focus groups did comment on how they had made changes to their behaviour outside working hours, for example, by getting up during the TV adverts, standing whilst engaging in screen-based activities and on the phone, and walking more. However, often participants said these were small changes and the intervention had made more impact during work, which aligns with the findings from the device-based measures [16]. Furthermore, others reported not changing their behaviour at home and often they felt tired after a work day and wanted to relax by sitting down. The setting-driven shifts in behaviour (i.e., that the majority of changes occurred at the work setting) are consistent with other workplace sedentary reduction interventions [31].

Participants reported perceiving several benefits from the intervention, with many of these clustered around feeling more energized and alert. These reinforce our quantitative findings of small improvements in vigor for both intervention groups [16]. Similar benefits were also reported in a review on perceptions of the feasibility and acceptability of reducing occupational sitting [19]. Our quantitative findings also demonstrated improvements in pain in the lower extremity for the SWAL plus desk group only [16] and this was also evident from our focus groups where participants in this group reported feeling less aches and pains. These benefits are consistent with those reported in our previous intervention, SMArT Work [27].

Work pressure has been identified as a barrier in previous interventions focused on reducing sitting at work when the intervention implementation is the responsibility of existing staff within an organisation [32]. SWAL workplace champions also reported work pressures as a barrier to implementing some of the strategies, despite organisational support for intervention delivery, via time allocation. This suggests that there may be some disconnect between how the organisational-level support was implemented and communicated, beyond the provision of time. Management support is identified as a key facilitator to workplace health promotion [33], with visible role modelling of desired behaviours by management seen as important for creating a permissive culture for change [34, 35]. If organisations invest in these types of programmes, and volunteers within the organisations are to be trained and used to facilitate health improvement initiatives, then organisations will need to ensure champions’ workload takes into consideration the time needed to implement the programme to maximise its effectiveness. Alternatively, the organisations will need to fund an external implementation partner. Champion characteristics (e.g., resilience, enthusiasm) have also been shown to have a role in implementation success [34, 36]. To inform future intervention implementation, research should investigate whether the characteristics of the champions differ and if this is associated with the level of intervention implementation, participant experiences and intervention effectiveness.

Participants in our previous intervention [27], and other interventions focused on reducing occupational sitting [19], reported forgetting to break up their sitting when busy with work, forgetting to use the height-adjustable desk in the standing position, feeling awkward whilst standing, charging of the sitting self-monitoring tool and lack of management buy-in as common barriers. SWAL tried to address these barriers through the provision of various self-monitoring and prompt tools that were freely available and did not require regular charging, motivational posters to display in the office and change monthly, social support through group catch up sessions and regular sitting less challenges, small environmental changes within the office to encourage more standing and movement, increased manager buy-in through providing manager education, manager role modelling, staff to volunteer as workplace champions and manager’s granting protected time each month for workplace champions. Despite this many of the same barriers to reducing and breaking up sitting still emerged that have been seen in the broader literature [19, 32, 37]. These include workload and work pressures (e.g., urgent work), the work environment being designed for sitting, feeling self-conscious when standing, not having a height-adjustable desk (SWAL only participants), tiredness, laziness and forgetting. Ultimately, creating a dynamic workplace (where less sedentary time and more movement is the norm) will likely require a culture change, with actions implemented at all levels of influence (organisational, environmental, intrapersonal, interpersonal). Understanding of change management and decision making processes, and integrating these concepts into the intervention design, will likely help in the acceptance, adoption and sustainability of the intervention [38].

It is important to acknowledge that the SWAL intervention and evaluation took place prior to the COVID-19 pandemic. Since then, as a result of the COVID-19 pandemic, working patterns and locations have changed, with many people working from home for all or some of their working week [39]. Therefore it will be important to understand whether the intervention strategies that were effective in reducing workplace sitting time are transferable to this new context. A recent study in University staff working predominantly from home suggests that intervention strategies should be aimed at encouraging regular breaks in sitting as well as enhancing automatic motivation and physical opportunity influences [40]. Furthermore, a review examining whether effective intervention strategies in an office environment to reduce sitting time could be transferable to the home working environment concluded that educational materials, role models, incentives, and regular prompts show promise for the home working environment [41]. This research suggests that many of the strategies included within SWAL may be transferable to the home working environment. It is clear however from the current study, and other previously successful workplace sitting reduction interventions [41], that having access to a height-adjustable desk is viewed as a key driver for behaviour change. Restructuring the physical environment and adding objects to the environment however was not deemed as transferable to the home environment in the recent review by Morton and colleagues [41], although some stakeholders did rate this as possibly transferable. Therefore, further research is needed to understand the acceptability and feasibility of the different intervention strategies for a home working environment.

### Strengths and limitations

The main strength of this process evaluation was the multiple mixed methods employed to gather data on engagement and experiences across the course of the study. There was good representation in the focus groups from the clusters, although we were unable to conduct focus groups in one of the smaller councils involved in the study. The majority (~ 80%) of participants and approximately half of champions provided quantitative data. The intervention was conducted in one type of industry only (council office workers). To understand the generalisability of the SWAL intervention it will be important to evaluate it across a range of industries, and with workers who work under hybrid and remote working arrangements.

## Conclusions

The overall experiences of the workplace champions and participants were positive but it was clear that there was variation in how intervention strategies were experienced, with some intervention strategies being perceived more useful (education, height-adjustable desk, group catch up sessions) than others (sitting less challenges, self-monitoring). Despite many participants reporting some changes at home, many felt the main changes to sitting behaviour were at work which explains why the device-measured sitting reductions were only observed during work hours in the trial. It is evident from these data that different intervention strategies will work for different people indicating that a ‘one size fits all’ approach may not be appropriate for this type of intervention, a finding also highlighted previously [16]. Identifying which intervention components bring about the greatest change would help to understand the core elements that are ‘mandatory’ and additional flexible elements that could be offered in a toolkit that participants and champions could ‘dip in and out of’ to suit them and their teams. The process evaluation data shows that the SWAL intervention could be tested in a broader range of organisations following a few minor changes including enhancing the workplace champion training to include role play of delivering group sessions, adding new content to the education session to use as a refresher/reminder, and providing champions with examples of how to adapt the intervention strategies (i.e., emails, group catch ups, challenges) to the needs of their group.

### Electronic supplementary material

Below is the link to the electronic supplementary material.

Supplementary Material 1

Supplementary Material 2

Supplementary Material 3

## Data Availability

The datasets used and/or analysed during the current study are available from the corresponding author on reasonable request.

## List of abbreviations

FG: Focus Group
SWAL: SMART Work and Life
WPC: Workplace Champion
