# Supplementary material for: Participant and workplace champion experiences of an intervention designed to reduce sitting time in desk-based workers: SMART work & life
Source: Int J Behav Nutr Phys Act. 2023 Nov 30;20:142. doi: 10.1186/s12966-023-01539-6 (PMC10691052; doi:10.1186/s12966-023-01539-6)
Supplement: Supplementary file 1 — Supplementary Material 1 [file 12966_2023_1539_MOESM1_ESM.docx]

|  | **Standards for Reporting Qualitative Research (SRQR)*** |  |
| --- | --- | --- |
|  | <http://www.equator-network.org/reporting-guidelines/srqr/> |  |
|  |  | **Page/line no(s).** |
| **Title and abstract** | |  |
|  | **Title** - Concise description of the nature and topic of the study Identifying the study as qualitative or indicating the approach (e.g., ethnography, grounded theory) or data collection methods (e.g., interview, focus group) is recommended | p1  Note: this is not a purely qualitative study; rather the paper reports on mixed methods that collectively continued to the process evaluation of the intervention. |
|  | **Abstract** - Summary of key elements of the study using the abstract format of the intended publication; typically includes background, purpose, methods, results, and conclusions | p3-4 |
|  |  |  |
| **Introduction** | |  |
|  | **Problem formulation** - Description and significance of the problem/phenomenon studied; review of relevant theory and empirical work; problem statement | p4-6 |
|  | **Purpose or research questio**n - Purpose of the study and specific objectives or questions | P5-6 lines 113-117 |
|  |  |  |
| **Methods** | |  |
|  | **Qualitative approach and research paradigm** - Qualitative approach (e.g., ethnography, grounded theory, case study, phenomenology, narrative research) and guiding theory if appropriate; identifying the research paradigm (e.g., postpositivist, constructivist/ interpretivist) is also recommended; rationale** | - Rationale for qualitative elements of process evaluation: p5 line 105-113 - Qualitative approach (in this case constant comparative strand of grounded theory, after Charmaz): p10 lines 225-226 |
|  | **Researcher characteristics and reflexivity** - Researchers’ characteristics that may influence the research, including personal attributes, qualifications/experience, relationship with participants, assumptions, and/or presuppositions; potential or actual interaction between researchers’ characteristics and the research questions, approach, methods, results, and/or transferability | - Characteristics of the researchers who generated the data through interviews and focus groups: p10 lines 217-220. - Characteristics of qualitative research lead (author HE) are not included in the manuscript currently, but could be added if the editor requests. |
|  | **Context** - Setting/site and salient contextual factors; rationale** | - Details of the (multiple) settings/sites: p6 lines 129-132, p 7 lines 143-150. - Rationale for settings (organisation type): p 5 lines 89-104 - Further rationale and details of sites are in the main RCT protocol and results papers, referenced in the current manuscript. |
|  | **Sampling strategy** - How and why research participants, documents, or events were selected; criteria for deciding when no further sampling was necessary (e.g., sampling saturation); rationale** | - Sampling strategy for main trial: p 6 lines 129-141 - Sampling strategy for workplace champion qualitative interviews: p6 lines 137-141 and p8 lines 185-193. - Sampling strategy for participant questionnaires: p9 lines 194-199 - Sampling strategy for participant focus groups: p9 lines 199-204 |
|  | **Ethical issues pertaining to human subjects** - Documentation of approval by an appropriate ethics review board and participant consent, or explanation for lack thereof; other confidentiality and data security issues | - Ethics approval: p6 lines 120-123; p42 lines 926-929. - Participant consent (for all study measurements, including interviews and focus groups): p 6 lines 122-123 |
|  | **Data collection methods** - Types of data collected; details of data collection procedures including (as appropriate) start and stop dates of data collection and analysis, iterative process, triangulation of sources/methods, and modification of procedures in response to evolving study findings; rationale** | Data collection methods:   - Workplace champion evaluation survey and questionnaire: p8 lines 185-191. - Workplace champion interviews p 8-9 lines 191-193; p9 lines 207-216. - Participant questionnaire: p9 lines 194-199. - Participant focus groups: p9 lines 199-222. |
|  | **Data collection instruments and technologies** - Description of instruments (e.g., interview guides, questionnaires) and devices (e.g., audio recorders) used for data collection; if/how the instrument(s) changed over the course of the study | Data collection instruments:   - Workplace champion evaluation survey and questionnaire: p8 lines 185-191. - Workplace champion interview topic guide p9 lines 207-212. - Participant questionnaire: p9 lines 194-199. - Participant focus group topic guide: p9 lines 207-216. |
|  | **Units of study** - Number and relevant characteristics of participants, documents, or events included in the study; level of participation (could be reported in results) | - Participant numbers and characteristics: p11-12 Table 1 and Supplementary Table 2 |
|  | **Data processing** - Methods for processing data prior to and during analysis, including transcription, data entry, data management and security, verification of data integrity, data coding, and anonymization/de-identification of excerpts | - p10 line 221-222; 233-234. |
|  | **Data analysis** - Process by which inferences, themes, etc., were identified and developed, including the researchers involved in data analysis; usually references a specific paradigm or approach; rationale** | - p10 lines 225-236. |
|  | **Techniques to enhance trustworthiness** - Techniques to enhance trustworthiness and credibility of data analysis (e.g., member checking, audit trail, triangulation); rationale** | - p10 lines 228-229; 234-241. |
|  |  |  |
| **Results/findings** | |  |
|  | **Synthesis and interpretation** - Main findings (e.g., interpretations, inferences, and themes); might include development of a theory or model, or integration with prior research or theory | - p11 lines 247-251; p33-34 lines 718-732 |
|  | **Links to empirical data** - Evidence (e.g., quotes, field notes, text excerpts, photographs) to substantiate analytic findings | - Data excerpts: p12-33 |
|  |  |  |
| **Discussion** | |  |
|  | **Integration with prior work, implications, transferability, and contribution(s) to the field -** Short summary of main findings; explanation of how findings and conclusions connect to, support, elaborate on, or challenge conclusions of earlier scholarship; discussion of scope of application/generalizability; identification of unique contribution(s) to scholarship in a discipline or field | - p33-40 |
|  | **Limitations** - Trustworthiness and limitations of findings | - p40 lines 892-899 |
|  |  |  |
| **Other** | |  |
|  | **Conflicts of interest** - Potential sources of influence or perceived influence on study conduct and conclusions; how these were managed | - p42 lines 934-945 |
|  | **Funding** - Sources of funding and other support; role of funders in data collection, interpretation, and reporting | - p42 lines 946-953 |
|  |  |  |
|  | *The authors created the SRQR by searching the literature to identify guidelines, reporting standards, and critical appraisal criteria for qualitative research; reviewing the reference lists of retrieved sources; and contacting experts to gain feedback. The SRQR aims to improve the transparency of all aspects of qualitative research by providing clear standards for reporting qualitative research. |  |
|  |  |  |
|  | **The rationale should briefly discuss the justification for choosing that theory, approach, method, or technique rather than other options available, the assumptions and limitations implicit in those choices, and how those choices influence study conclusions and transferability. As appropriate, the rationale for several items might be discussed together. |  |
|  |  |  |
|  | **Reference:** |  |
|  | O'Brien BC, Harris IB, Beckman TJ, Reed DA, Cook DA. **Standards for reporting qualitative research: a synthesis of recommendations.** *Academic Medicine*, Vol. 89, No. 9 / Sept 2014  DOI: 10.1097/ACM.0000000000000388 |  |
|  |  |  |
|  |  |  |
