# Supplementary material for: Participant and workplace champion experiences of an intervention designed to reduce sitting time in desk-based workers: SMART work & life
Source: Int J Behav Nutr Phys Act. 2023 Nov 30;20:142. doi: 10.1186/s12966-023-01539-6 (PMC10691052; doi:10.1186/s12966-023-01539-6)
Supplement: Supplementary file 2 — Supplementary Material 2 [file 12966_2023_1539_MOESM2_ESM.docx]

**Supplementary Table 1. Summary of intervention strategies (shading indicates when the strategy happened)**

|  | **Month** | | | | | | | | | | | |
| --- | --- | --- | --- | --- | --- | --- | --- | --- | --- | --- | --- | --- |
|  | **1** | **2** | **3** | **4** | **5** | **6** | **7** | **8** | **9** | **10** | **11** | **12** |
| **Organisational strategies** | | | | | | | | | | | | |
| Support from Senior management team |  |  |  |  |  |  |  |  |  |  |  |  |
| Management agreed protected time for workplace champions |  |  |  |  |  |  |  |  |  |  |  |  |
| Workplace champion 3 hour training |  |  |  |  |  |  |  |  |  |  |  |  |
| Manager online education |  |  |  |  |  |  |  |  |  |  |  |  |
| **Group and individual strategies** | | | | | | | | | | | | |
| Online education for individual staff (inc health consequences of siting too much, self-monitoring, goal setting, barrier identification, action planning) |  |  |  |  |  |  |  |  |  |  |  |  |
| Monthly emails^a^ |  |  |  |  |  |  |  |  |  |  |  |  |
| Self-monitoring and prompts^b^ |  |  |  |  |  |  |  |  |  |  |  |  |
| Sitting less and moving more challenges^c^ |  |  |  |  |  |  |  |  |  |  |  |  |
| Group catch up sessions^d^ |  |  |  |  |  |  |  |  |  |  |  |  |
| **Environmental strategies** | | | | | | | | | | | | |
| Small scale restructuring in the office inc standing meetings, high tables |  |  |  |  |  |  |  |  |  |  |  |  |
| Home environment |  |  |  |  |  |  |  |  |  |  |  |  |
| Workplace champion behavioural modelling |  |  |  |  |  |  |  |  |  |  |  |  |
| Motivational posters displayed^e^ |  |  |  |  |  |  |  |  |  |  |  |  |
| Height-adjustable workstation with guidance booklet (SWAL + Desk group only^f^ |  |  |  |  |  |  |  |  |  |  |  |  |

^a^ Examples of email content included: reminder of key messages from online education, activating your muscles, getting the balance right between sitting, standing and moving, top tips to reduce and break up sitting, self-monitoring and prompts, how much do you sit; ^b^ The participants had access tools that were free to access: Phone apps included Rise & Recharge, Sitting Timer, My Health Avatar. Computer software included Workrave, Varidesk Desktop App. Google Chrome Extension included Outstanding; ^c^ Stand for the height of the Top 10 tallest buildings in the world (1 metre = 1 minute of standing), Stand for the height of the Top 3 highest mountains in the world, Walk from Leicester to London (*1 mile = 2112 steps); ^d^* Content included: ‘What’s been going well and not so well’, ‘Facilitators to reducing sitting time’, ‘Strategies used to reduce sitting’, ‘Barriers’, ‘Reminder about online education resources’, ‘Review of key messages of online education’, *‘*What could you do as a group at work or changes that you could make in the office to reduce your sitting time’, ‘What could you do outside of work?’, ‘Reminder of the computer software suggested on the online education programme can be installed on home computers and laptops.’ and ‘Goal setting’.; e Example of poster message include: ‘Sit less than 50% of your day & move every 30 minutes’, ‘Stand up regularly to feel more energised and focused’, ‘Health benefits of sitting less’, ‘Fire your body back into action’, ‘Getting the balance right’, ‘What daily tasks could you do standing up’;^f^ Participants were able to select their preferred desk type and colour (black or white) from four models: Deskrite 100 (Posturite, Berwick, UK), Yo-Yo Desk Mini, Yo-Yo Desk 90, or Yo-Yo Desk Go (Sit-Stand Trading, Swindon, UK). All the desks were designed to sit on top of the participants’ existing workstation.

**Supplementary Table 2. Focus group representation by intervention arm and council**

| **Council** | **SWAL only** | | | **SWAL plus Desk** | | |
| --- | --- | --- | --- | --- | --- | --- |
|  | **Participant N** | **Percentage of arm** | **Clusters represented** | **Participant N** | **Percentage of arm** | **Clusters represented** |
| **1** | 20 | 23% | 82% | 27 | 28% | 80% |
| **2** | 8 | 32% | 75% | 5 | 23% | 67% |
| **3** | 10 | 33% | 75% | 11 | 48% | 100% |
| **4** | 6 | 20% | 60% | 5 | 13% | 50% |
| **5** | 5 | 25% | 100% | 3 | 60% | 100% |
| **6** | 0 | 0% | 0% | 0 | 0% | 0% |
| **Total** | 49 | 25% | - | 51 | 24% | - |

**Supplementary Table 3. Workplace champion characteristics**

|  | **All workplace champions recruited** | | **Workplace champions who provided questionnaire data** | | **Workplace champions who were interviewed** | |
| --- | --- | --- | --- | --- | --- | --- |
|  | **SWAL only (n=27 clusters)** | **SWAL plus Desk (n=25 clusters)** | **SWAL only** | **SWAL plus Desk** | **SWAL only** | **SWAL plus Desk** |
| **n** | 27 | 29 | 14 | 13 | 9 | 7 |
| **Age** | 42.9 (11.1) | 43.5 (11.0) | 44.6 (11.5) | 46.1 (12.4) | 49.6 (8.1) | 47.0 (11.8) |
| **Sex, n female (%)** | 22 (81%) | 20 (69%) | 11 (79%) | 9 (69%) | 7 (78%) | 6 (86%) |
| **Ethnicity, n white British (%)** | 27 (100%) | 24 (83%) | 14 (100%) | 11 (85%) | 9 (100%) | 5 (71%) |
| **Duration of work at the council (yrs)** | 10.3 (8.6) | 12.3 (9.7) | 12.6 (10.2) | 12.9 (11.4) | 15.6 (9.8) | 17.3 (12.9) |
| **Manage staff, n yes (%)** | 7 (26%) | 10 (34%) | 5 (35%) | 3 (23%) | 4 (44%) | 2 (29%) |

**Supplementary Table 4. Participant characteristics for those who completed the process evaluation questionnaire**

|  | **3 Months** | | **12 Months** | |
| --- | --- | --- | --- | --- |
|  | **SWAL only** | **SWAL plus Desk** | **SWAL only** | **SWAL plus Desk** |
| **n** | 179 | 189 | 171 | 163 |
| **Age** | 44.1 (9.7) | 46.4 (9.9) | 43.3 (9.7) | 46.1 (10.1) |
| **Sex, n female (%)** | 130 (72.6%) | 126 (66.7%) | 126 (73.7%) | 111 (68.1%) |
| **Ethnicity, n white British (%)** | 132 (73.7%) | 139 (73.5%) | 118 (69.0%) | 119 (73.0%) |
| **Duration of work at the council (yrs)** | 12.1 (9.0) | 13.3 (9.5) | 11.9 (9.2) | 13.1 (9.6) |
| **Manage staff, n yes (%)** | 67 (37.4%)) | 78 (41.3%) | 62 (36.3%) | 61 (37.4%) |

**Supplementary Table 5. Participant characteristics for those who took part in the focus groups**

|  | **SWAL only** | **SWAL plus Desk** |
| --- | --- | --- |
| **n** | 49 | 51 |
| **Age** | 46.5 (9.1) | 47.3 (9.7) |
| **Sex, n female (%)** | 33 (67.3%) | 38 (66.7%) |
| **Ethnicity, n white British (%)** | 35 (71.4%) | 37 (64.9%) |
| **Duration of work at the council (yrs)** | 13.2 (9.0) | 13.0 (9.8) |
| **Manage staff, n yes (%)** | 14 (28.6%) | 21 (36.8%) |
