# Supplementary material for: Participant and workplace champion experiences of an intervention designed to reduce sitting time in desk-based workers: SMART work & life
Source: Int J Behav Nutr Phys Act. 2023 Nov 30;20:142. doi: 10.1186/s12966-023-01539-6 (PMC10691052; doi:10.1186/s12966-023-01539-6)
Supplement: Supplementary file 3 — Supplementary Material 3 [file 12966_2023_1539_MOESM3_ESM.docx]

Group catch ups with workplace champions

Manager agreed champion protected time

Provision for activity during work e.g., walking meetings, competitions

Workplace champion support

Management education

Motivational signs

Environmental restructuring: reallocation of printers, bins, standing meeting rooms

Choice of height-adjustable workstation, training and leaflet

Online education

Action planning, goal setting, barrier identification and solutions

Self-monitoring and prompting with free apps, computer software

The intervention is grounded in several behaviour change theories and implemented through the Behaviour Change Wheel (BCW) intervention functions

Organisational: Grounded in Social Cognitive Theory (SCT) and Organisational Development Theory (ODT). BCW intervention functions: enablement, persuasion, environmental restructuring, modelling

Environmental: Grounded in SCT, ODT and Habit Theory. BCW intervention functions: environmental restructuring, enablement and training

Individual and Group: Grounded in SCT, Self-Regulation Theory and Relapse Prevention Theory. BCW intervention functions: enablement, persuasion, education and training

Senior management role modelling, encouragement

Enhanced self-regulation skills. Real time feedback on sitting levels and real time prompts to change posture regularly leads to reductions in sitting time

Enhanced knowledge of the risks of sitting too much and prolonged sitting, benefits of reducing and breaking up sitting time

More opportunity for standing/moving throughout the day leads to reductions in sitting time

Increased social interaction and peer support

Enhanced group motivation and encouragement

**Short term outputs/goals**

Reductions in overall sitting time and prolonged sitting sustained

Improved physiological health (reduction in musculoskeletal issues, improvements in adiposity and glucose and cholesterol of office workers

Improved psychological health (anxiety, depression, stress) and well-being (quality of life) of office workers

Improvements in workplace outcomes (absenteeism, work engagement, job performance and satisfaction, presenteeism)

Interventions are cost-effective

**Long term outputs/goals**

**Available resources and activities**

**Underpinning model and theories**
